# Inflammation of the rectal remnant endangers the outcome of ileal pouch-anal anastomosis: a case–control study

**DOI:** 10.1007/s00384-022-04195-7

**Published:** 2022-06-17

**Authors:** Jan P. Frese, Jörn Gröne, Johannes C. Lauscher, Martin E. Kreis, Benjamin Weixler, Katharina Beyer, Claudia Seifarth

**Affiliations:** 1 Department of General, Visceral, and Vascular Surgery, Charité – Universitätsmedizin Berlin, corporate member of Freie Universität Berlin, Humboldt-Universität Zu Berlin, and Berlin Institute of Health, Berlin, Germany; 2 Department of General and Visceral Surgery, St. Joseph Krankenhaus, Berlin, Germany

**Keywords:** Ulcerative colitis, Ileal pouch-anal anastomosis, IPAA, Inflammatory bowel disease, Proctitis, Pouchitis, Pouch failure

## Abstract

**Background:**

Inflammation of the rectal remnant may affect the postoperative outcome of ileal pouch-anal anastomosis (IPAA) in patients with ulcerative colitis (UC). We aimed to determine the extent of inflammation in the anastomotic area during IPAA and to investigate the impact of proctitis on postoperative complications and long-term outcomes.

**Methods:**

Three hundred thirty-four UC patients with primary IPAA were included in this retrospective case–control study. The histopathologic degree of inflammation in the anastomotic area was graded into three stages of no proctitis (“NOP”), mild to medium proctitis (“MIP”), and severe proctitis (“SEP”). Preoperative risk factors, 30-day morbidity, and follow-up data were assessed. Kaplan–Meier analysis was performed in the event of pouch failure.

**Results:**

The prevalence of proctitis was high (MIP 40.4%, and SEP 42.8%). During follow-up, the incidence of complications was highest among SEP: resulting in re-intervention (*n* = 40; 28.2%, *p* = 0.017), pouchitis (*n* = 36; 25.2%, *p* < 0.01), and pouch failure (*n* = 32; 22.4%, *p* = 0.032). The time interval to pouch failure was 5.0 (4.0–6.9) years among NOP, and 1.2 (0.5–2.3) years in SEP (*p* = 0.036). ASA 3, pouchitis, and pouch fistula were independent risk factors for pouch failure.

**Conclusion:**

Proctitis at the time of IPAA is common. A high degree of inflammation is associated with poor long-term outcomes, an effect that declines over time. In addition, a higher degree of proctitis leads to earlier pouch failure.

## Introduction

Restorative proctocolectomy with ileal pouch-anal anastomosis (IPAA) is the gold standard in the surgical treatment of ulcerative colitis [1]. According to most guidelines, the procedure is typically performed in several stages in patients with long-term pharmacological immunosuppression, reduced general condition, and nutritional status [2]. In the three-staged procedure, a colectomy with a rectal stump and terminal ileostomy is performed first. After a few months of recovery, the second step, namely proctectomy and the creation of the ileal pouch, is performed. Some patients develop or retain proctitis in the remaining rectal stump after colectomy [3]. Little is known about the actual mid- and long-term consequences of proctitis on the outcome of IPAA. Proctitis can be treated conservatively with local anti-inflammatory therapy, but often does not heal by the time of IPAA [2]. The exact causes of proctitis are unclear. There are data indicating a higher rate of anastomotic leakage after IPAA surgery in the case of proctitis [4]. However, these results are based on a small study in pediatric surgery. There is also a suggestion that the presence of colitis or proctitis is associated with a higher rate of pouchitis [3, 5] or a higher failure rate of the IPAA [6]. A rate of up to 30% of chronic pouchitis has been reported [5]. This study aimed to investigate the extent of inflammation in the anastomotic area during IPAA and to determine the impact of perioperative proctitis on postoperative complications and long-term outcomes. We hypothesized that a higher degree of inflammation in the rectal remnant during IPAA compromises the success of the procedure.

## Methods

### Study cohort and design

**Fig. 1:**
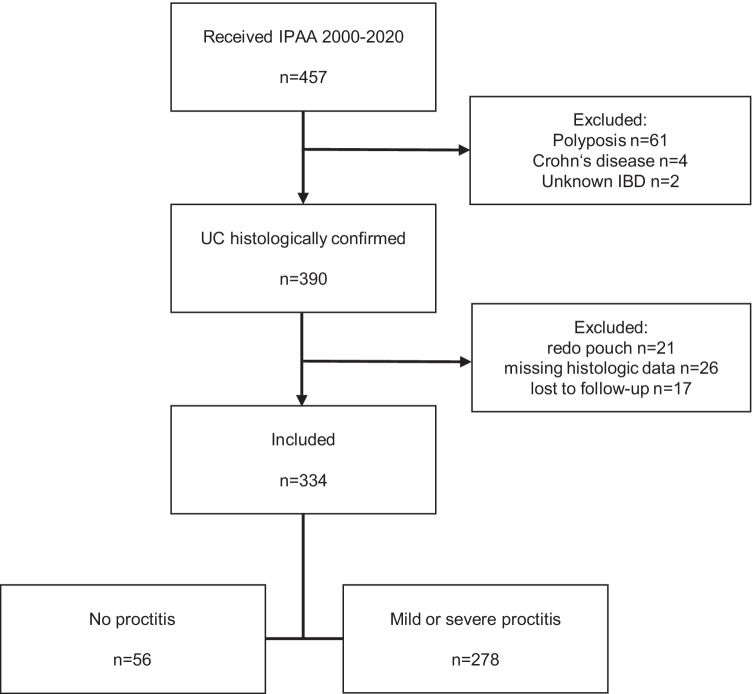
Flowchart of the study selection process. IPAA: ileal pouch-anal anastomosis. UC: ulcerative colitis. IBD: inflammatory bowel disease. Note: for several cases, more than one exclusion criterion applied

A prospectively maintained database containing the records of 457 patients who had received IPAA in our tertiary referral center between 2000 and 2020 was screened for study participation. Of those, 390 (85.3%) patients were diagnosed with UC. Figure 1 depicts the study selection process. Exclusion criteria were redo pouch (*n* = 31) (secondary pouch construction after removal of a failed IPAA), insufficient histopathologic (*n* = 26), or follow-up data (*n* = 17). Three hundred thirty-four UC patients with primary IPAA and sufficient documentation were included in this retrospective case–control study. The results of the study were reported according to the “Strengthening the Reporting of Observational Studies in Epidemiology” (STROBE) guidelines [7]. The institutions’ medical ethical committee approved this study (EA4/195/20). The need for individual written informed consent was waived.

### Surgical procedures, complications, and follow-up

All two- and three-staged IPAA procedures were included. In patients exhibiting a high activity of UC, under high-dose of steroid immunosuppression, reduced general condition, and nutritional status, or in all emergency procedures, a three-staged approach was chosen [8, 9]. The first stage was colectomy, end-ileostomy, and terminal sigmoidostomy or blind closure of the rectal stump. The ileo-anal pouch was created several months later when the patients had recovered and immunosuppression could have been stopped. According to newer guidelines’ recommendations, the interval between the first and second step was shorter in the late years of the study [8]. In cases of clinically low activity of UC, dysplasia or cancer, a two-staged approach was preferred, with the proctocolectomy and pouch-anal anastomosis being performed simultaneously [9]. An ileo-ileal J-pouch configuration and a diverting loop ileostomy were used in all cases. IPAA was created either by double-stapling technique or transanal hand suture. During the 20 years of the study, a paradigm shift occurred from hand-sewn to stapled anastomoses and open to laparoscopic procedures. Postoperative complications within 30 days after IPAA were scored according to the Classification of Clavien and Dindo [10]. A score of 2 or greater than 2 was considered to be a relevant surgical complication. After discharge, all patients were invited to a structured follow-up at our institution, which consisted of an interview, clinical examination, and flexible endoscopy of the pouch. Follow-up appointments were scheduled at 3, 6, and 12 months and yearly thereafter. Patients who did not adhere to scheduled appointments, who could not be contacted by proxy, and non-survival could not otherwise be confirmed, were considered as lost to follow-up and excluded from the study. Incidence and risk factors of pouch failure were evaluated over time. Pouch failure was defined as the need for a secondary diverting or terminal ileostomy with or without pouch explantation, thus including loss of pouch function with pouch remaining in situ in some cases. The 5-year interval was considered as the threshold between “mid-term” and “long-term” results [11].

### Histological assessment

All patients included in the study had a complete histologic workup of their resected bowel specimens. In two-staged IPAA, the area of the distal resection margin of the proctocolectomy specimen was considered as the region of interest for this study. In three-staged IPAA, the rectal remnant that was resected before IPAA creation during the second step was analyzed. Several scoring systems for histologic chronicity and activity of UC had been developed, such as the Geboes score [12], or more recently, the Nancy index [13]. Two pathologists assessed the disease-specific inflammation in general and diversion-induced inflammation in three-staged IPAA. In our study, the actual degree of active inflammation was considered to be the most relevant parameter for IPAA outcome. Based on Florén et al. [14] and the later simplified Mount Sinai system by Gupta et al. [15], the degree of disease-specific acute inflammation was graded into three stages: no proctitis (NOP group), mild to medium proctitis (MIP group), or severe proctitis (SEP group).

### Variables and statistics

In this retrospective study, clinical data, postoperative complications, and follow-up parameters were collected from a prospective patient database. Statistical analysis was performed with IBM SPSS, v27 (IBM, Armonk NY, USA). Unless otherwise indicated, measures of location and variability are reported as the median and interquartile range (IQR), as most of the continuous variables were not normally distributed in Shapiro–Wilk tests. Categorical variables were compared by means of chi-square tests or with Fisher’s exact test in cases with group size < 5. Continuous outcome variables were compared by a nonparametric Kruskal Wallis test. A statistically significant difference was assumed for a *p* value < 0.05, marked with two asterisks**. A *p* value < 0.1 was considered a trend and marked with one asterisk*. Multivariate analysis was performed through logistic regression. Covariates were included in the model stepwise using forward selection based on the likelihood ratio and the Akaike information criterion. Kaplan–Meier analysis and log-rank tests were used to compare the degree of inflammation and pouch survival.

## Results

### Patients and interventions

Three hundred thirty-four UC patients with primary IPAA were included in this study. The study selection process is depicted in Fig. 1. The indications for surgery were pharmacologically refractory disease (*n* = 235; 70.4%), colorectal cancer or high-grade dysplasia (*n* = 68; 20.4%), and emergency events (ileus, bowel perforation, toxic megacolon, bleeding) (*n* = 31; 9.3%). One hundred eighty patients (53.9%) were male, the median age at the time of IPAA was 36.8 (29.2–48.1) years. The portion of three-staged operations was 62.9% (*n* = 210). In three-staged IPAA, the subtotal colectomy took place 5.0 (3.0–8.0) months prior to the IPAA. There were 234 (70.1%) laparoscopic procedures, and hand-sewn anastomoses were created in 179 cases (53.6%). During the late years of the study, most IPAA were stapled anastomoses. Epidemiologic and procedural details are given in Table 1.

**Table 1:** Anthropometric characteristics and procedural details of the 334 cases included, at the time of the restorative proctocolectomy (IPAA). Categorical variables are given as numbers with portions in brackets. Metric variables are shown as median and interquartile range in brackets

Male sex		180	(53.9)
Age at diagnosis [years]		26.7	(20.9–35.9)
Age at IPAA [years]		36.8	(29.2–48.1)
Age > 50 years		73	(21.9)
Indication for surgery			
Refractory disease		235	(70.4)
CRC or high-grade IEN		68	(20.4)
Emergency		31	(9.3)
Degree of distal inflammation			
No proctitis		55	(16.5)
Mild proctitis		136	(40.7)
Severe proctitis		143	(42.8)
Diabetes		28	(8.4)
Hypertension		41	(12.3)
Extraintestinal manifestation of UC		84	(25.1)
Cushing's Syndrome		17	(5.1)
Active malignancy		43	(12.9)
Body mass index (BMI) [kg/m^2^]		23.3	(20.2–25.9)
BMI > 27.5		56	(16.8)
ASA category	1	15	(4.5)
	2	281	(84.1)
	3	38	(11.4)
**Medication**			
Biologics		94	(28.1)
Prednisolone		64	(19.2)
Azathioprine		16	(4.8)
Topical steroids or 5-ASA		48	(14.4)
**Details of IPAA procedure**			
Anastomosis	Handsewn	179	(53.6)
	Stapled	155	(46.4)
Staged IPAA	Three-stage	210	(62.9)
Three-stage: interval since colectomy [months]		5.0	(3.0–8.0)
Laparoscopic IPAA		234	(70.1)

*CRC* colorectal cancer, *IEN* intra-epithelial neoplasia, *UC* ulcerative colitis, *ASA* American Society of Anesthesiologists physiological classification

### Histopathological findings and IPAA outcome

The histopathologic degree of inflammation was determined in the rectal remnant of all 210 three-staged IPAA, and in the distal resection area of the remaining 124 IPAA. No inflammation (“NOP group”) was found in 56 (16.8%) cases; mild to medium proctitis (“MIP group”) was found in 135 (40.4%), and severe proctitis (“SEP group”) in 143 cases (42.8%). The portion of three-staged IPAA was 51.8% in NOP, 57.0% in MIP, and 72.7% in SEP (*p* < 0.01).

Prior to IPAA, the rate of patients that were treated with rectal local steroids or 5-ASA was 20.3% in SEP and only 12.5% in NOP, respectively (*p* = 0.023). The 30-day morbidity of NOP, MIP, and SEP regarding Clavien-Dindo score, and specific surgical complications (anastomotic leakage, postoperative ileus, and urinary tract complication) did not differ between the three different proctitis groups, as you can see in Table 2. During the median follow-up of 3.9 (0.7–5.8) years, the incidence of complications was highest among SEP: patients requiring re-intervention (*n* = 40; 28.2%, *p* = 0.017), pouchitis (*n* = 36; 25.2%, *p* < 0.01), and pouch failure (*n* = 32; 22.4%, *p* = 0.032). The time interval to pouch failure (“pouch survival time”) was 5.0 (4.0–6.9) years among NOP and only 1.2 (0.5–2.3) years in SEP (*p* = 0.036). Details of univariate group comparisons of the outcome parameters are described in Table 2.

**Table 2:** Univariate group comparisons of periprocedural and long-term complications of the study cohort between three subgroups of inflammation severity. Categorical variables are given as numbers with proportions in brackets. Metric variables are shown as median and interquartile range in brackets

	**Prevalence**		**No proctitis (NOP)**		**Mild proctitis (MIP)**		**Severe proctitis (SEP)**		***p* value**
Total cases	334	(100)	56	(16.8)	135	(40.4)	143	(42.8)	
Three-stage IPAA	210	(62.9)	29	(51.8)	77	(57.0)	104	(72.7)	< 0.01*
**30-day morbidity**									
Clavien-Dindo score									0.132
II	29	(8.7)	1	(1.8)	14	(10.3)	14	(9.8)	
IIIa	17	(5.1)	2	(3.6)	7	(5.1)	8	(5.6)	
IIIb	39	(11.7)	6	(10.7)	15	(11.1)	18	(12.6)	
IVa	10	(3.0)	1	(1.8)	3	(2.2)	6	(4.2)	
IVb	3	(0.9)	1	(1.8)	0	(0)	2	(1.4)	
V	1	(0.3)	1	(1.8)	0	(0)	0	(0)	
Leakage, early fistula	52	(15.6)	8	(14.5)	19	(14.0)	25	(17.5)	0.702
Urinary tract complication	28	(8.4)	3	(5.5)	12	(8.8)	13	(9.1)	0.690
Prolonged ileus	47	(14.1)	9	(16.4)	17	(12.5)	21	(14.7)	0.755
**Follow-up**									
Follow-up interval [years]	3.9	(0.7–5.8)	4.6	(0.5–7.8)	3.1	(0.5–4.0)	4.4	(1.0–7.4)	0.056
Requiring re-Intervention	70	(21.0)	7	(12.7)	23	(16.9)	40	(28.2)	0.017*
Secondary ileostomy	28	(8.4)	4	(7.3)	9	(6.6)	15	(10.5)	0.480
Pouchitis	65	(19.5)	14	(25.5)	15	(11.0)	36	(25.2)	< 0.01*
IPAA stenosis	42	(12.6)	8	(14.5)	13	(9.6)	20	(14.1)	0.378
Fistula	37	(11.1)	3	(5.5)	14	(10.3)	20	(13.9)	0.208
Incontinence	28	(8.4)	4	(7.3)	12	(8.8)	12	(8.5)	0.940
Pouch explantation	21	(6.3)	3	(5.5)	5	(3.7)	13	(9.2)	0.165
Pouch failure [n]	55	(16.5)	5	(9.1)	18	(13.2)	32	(22.4)	0.032*
Pouch survival time [years]	3.1	(0.8–3.6)	5.0	(4.0–6.9)	2.1	(1.4–2.5)	1.2	(0.5–2.3)	0.036*

*IPAA* ileal pouch-anal anastomosis

*indicates *p* value < 0.05

### Univariate and multivariate risk factors for pouch failure

Univariate risk factors (*p* < 0.05) for pouch failure during a 10-year follow-up after IPAA were ASA category 3, a severe degree of inflammation at the time of IPAA (SEP), pouchitis, and pouch fistula. In the multivariate regression, ASA, pouchitis, and pouch fistula were included in the model as significant independent variables (*p* < 0.01). Severe proctitis was not included in the model because of a significant degree of collinearity with pouchitis (*r* = 0.146, *p* = 0.007). The hazard ratios and the 95% confidence intervals can be found in Table 3.

**Table 3:** Risk factors with hazard ratios for 10-year pouch failure, univariate und multivariate analysis

	*n* (%)	Univariate (95% CI)		*p* value	Multivariate (95% CI)		*p* value
Pouch failure (10 years)	52 (100)						
Male	24 (46.2)	0.816	(0.596–1.116)	0.180			
Kidney disease	5 (9.6)	2.651	(0.944–7.445)	0.058			
Extraintestinal manifestation	18 (34.6)	1.446	(0.940–2.224)	0.107			
ASA category 3	15 (28.8)	3.485	(1.935–6.179)	< 0.01*	4.596	(2.113–9.996)	< 0.01*
Severe proctitis (at IPAA)	32 (61.5)	1.501	(1.157–1.948)	< 0.01*			
Pouchitis	19 (36.5)	2.190	(1.401–3.423)	< 0.01*	2.211	(1.105–4.422)	0.025*
Pouch fistula	17 (32.7)	4.593	(2.585–8.162)	< 0.01*	6.233	(2.895–13.419)	< 0.01*

*ASA* American Society of Anesthesiologists physiological classification, *IPAA* ileo-pouch-anal anastomosis, *CI* confidence interval

*indicates *p* value < 0.05

### Proctitis and pouch survival

**Fig. 2:**
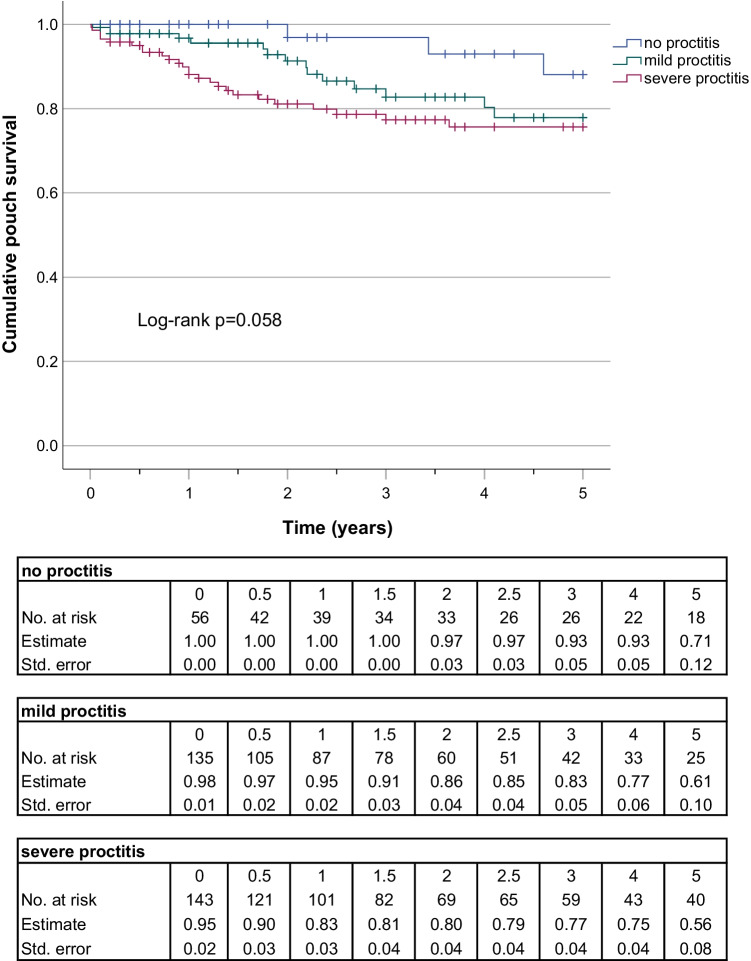
Kaplan‐Meier curve and lifetables of 5-year pouch survival rate before pouch failure in patients with no inflammation, mild, or severe inflammation in the rectal remnant at the time of restorative proctocolectomy and pouch creation

**Fig. 3:**
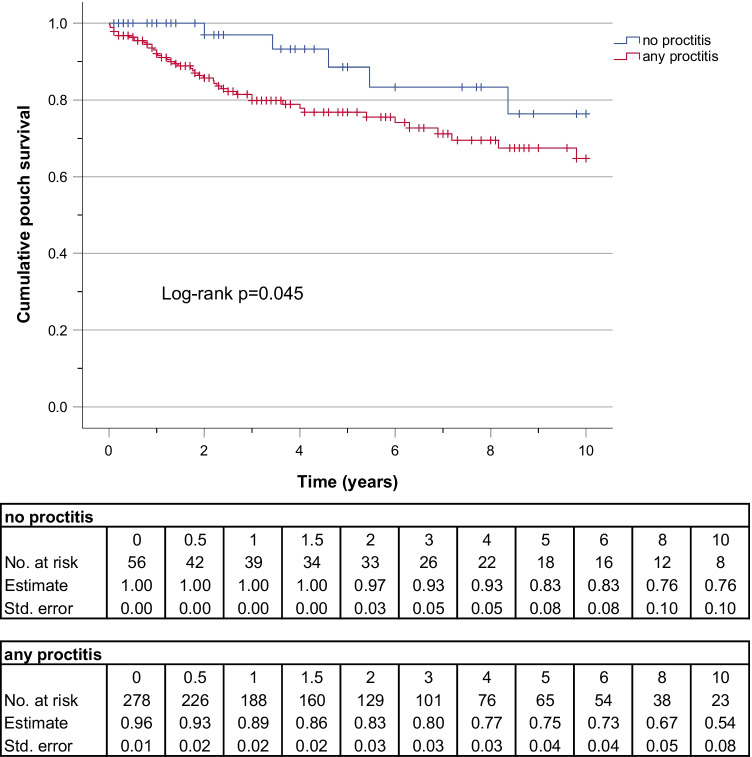
Kaplan‐Meier curve and lifetables of 10-year pouch survival rate before pouch failure in patients with or without inflammation in the rectal remnant at the time of restorative proctocolectomy and pouch creation

The Kaplan Meier (KM) curves and life tables of 5- and 10-year pouch survival rates are depicted in Figs. 2 and 3. The rate of pouch survival was compared between the three groups of NOP, MIP, and SEP. The KM-curve of pouch survival was declining fastest in the severe proctitis group. After 5 years, however, no difference could be found between MIP and SEP; thus, the log-rank test was not significant (*p* = 0.058) (Fig. 2). Therefore, for the 10-year analysis, the MIP and SEP groups were pooled and only two groups “no proctitis” and “any degree of proctitis” were compared. The rate of pouch survival was lower in the second category (log-rank *p* = 0.045) (Fig. 3); however, the gap was closing over time.

## Discussion

Restorative proctocolectomy is the gold standard in the definitive treatment of refractory UC and UC associated colorectal cancer, including its preliminary states [16]. Determined by the pathophysiology of UC, the disease activity is highest in the distal colon and rectum, the area where IPAA is performed [2]. The integrity of IPAA is paramount for postoperative complications and long-term success including quality of life and continence [17, 18]. The study aimed to investigate the prevalence of inflammation in the anastomotic area during IPAA and to determine the impact of proctitis on postoperative complications and long-term outcomes. We hypothesized that a higher degree of inflammation during IPAA compromises the future success of the procedure.

In this retrospective, single-center study, we showed that rectal stump inflammation or inflammation in the distal anastomotic area is common, with mild to medium degree of proctitis (MIP) in 40.4% and severe proctitis (SEP) in 42.8 of our cases. This high rate of proctitis is similar to the rate found in one other recent study [3]. In that study, no differentiation was made into several degrees of inflammation. This is important because we could show that a higher degree of inflammation leads to more severe consequences. With an increasing degree of proctitis, the portion of three-staged IPAA was rising. This fits the recommendations of the guidelines [9]. The choice for a three-staged concept in cases with a severe UC and high-dose immunosuppression results in fewer perioperative complications and better long-term outcomes [18–20]. We interpreted the higher degree of proctitis in three-staged IPAA as an indicator of the overall higher activity of the disease in those patients, which led to the decision for a three-staged concept. We could show that proctitis is a risk factor for complications during follow-up after IPAA. The idea that local inflammation in the rectal stump may influence the fate of IPAA, even after complete resection of the diseased and inflammation-bearing rectal mucosa, is not yet well established, and the mechanism is not clear. In three-staged interventions, the inflammation of the rectal stump could trigger pelvic abscesses [21]. In addition, it is known that residual rectal mucosa after IPAA triggers long-term complications [22]. It may be speculated that this is the reason for complications related to pouchitis, but there may also be an effect of local inflammation on the surrounding tissues in the deep pelvis. This uncertainty may be one reason for the low rate of patients receiving topical anti-inflammatory medication. This rate was higher in severe proctitis compared to NOP (20.3% vs. 12.5%, *p* = 0.023), but still, only every 5th of SEP patients had received this therapy, similar to the study of Wasmann et al. [3]. There is evidence that 5-ASA enemas improve the activity of inflammation in UC [23]. Topical treatment of UC shows good results with low side effects according to a recent review in non-surgical UC patients under conservative treatment [24]. Furthermore, the endoscopic view may underestimate the histologic extent of inflammation [25]. To our best knowledge, there is no study or guideline aiming to optimize the local inflammation in surgical patients prior to IPAA. This conflicts with the high rate of proctitis and the rate of complications associated with proctitis after IPAA.

Regarding postoperative complications, there is one study showing no influence of proctitis on the rate of anastomotic leakage [4]. In our study, the 30-day morbidity was not different between the degrees of proctitis. During long-term follow-up, the rate of re-interventions, pouchitis, and pouch failure was significantly rising with the degree of inflammation at the time of IPAA. The time to pouch failure was shorter in severe proctitis. ASA 3, pouchitis, and pouch fistula were identified as independent predictors for pouch failure. In a recent retrospective study regarding pouch failure [26], the overall failure rate was lower than in our study (15.5% vs. 16.5%), but their definition of failure included only pouch explantation. Pouchitis and pouch fistula were independent risk factors, too.

Pouch survival was shortest in severe proctitis; however, after 5 years, there was no difference to medium proctitis. After 10 years, there still was a difference in pouch survival between the groups with any degree of inflammation and with no proctitis, but the gap was closing over time. As in our study, the failure rate was highest during the first year in a large Cleveland Clinic study [27]. From a clinical perspective, it may be reasonable to assume that the impact of proctitis at the time of IPAA on follow-up complications is highest in the first 5 years (“mid-term”). As other factors may become more important for pouch failure during the 10-year period, the influence of proctitis is declining over time. It seems plausible that a higher degree of inflammation causes more pouch-related complications in the first years. In the later course, the influence of proctitis diminishes.

One limitation of the study is the relatively low number of pouch failures (*n* = 5) in the group without proctitis, raising the question of model stability. Due to the retrospective design, it cannot be shown if there is a beneficial effect of topical anti-inflammatory treatment or just a correlation. A weakness of the study’s design is that in three-staged IPAA, the influence of diversion colitis could not be determined exactly. A strength of the study is the single-center design in a tertiary referral center for inflammatory bowel disease with a homogenous way of diagnosing and treating UC. A possible implication for future studies could be the re-evaluation of the degree of proctitis and prescription of local anti-inflammatory treatments some time before IPAA, in order to evaluate a benefit for IPAA results.

## Conclusion

Proctitis at the time of IPAA is associated with poor long-term outcomes, an effect that declines over time. In addition, a higher degree of proctitis leads to earlier pouch failure. This is caused by high comorbidity and inflammation of the pouch. There is no study or guideline addressing the question of preoperative optimization by topical enemas or suppositories. Further prospective studies are needed to evaluate the benefit of an intensified preoperative local anti-inflammatory therapy prior to IPAA.

## Data Availability

The data collection was done by reviewing the electronic health records at the Charité Universitätsmedizin Berlin. Relevant data were collected in Microsoft Excel and analyzed descriptively. The data are stored on the Charité server, and in order to avoid a violation of access rights, the data are encrypted using a password only known to the study physicians. The data that support the findings of this study are available, but restrictions apply to the availability of these data, which were used under license for the current study, and so are not publicly available. Data are however available from the authors upon reasonable request.
